# Katharina Kreuder-Sonnen 2018: Wie man Mikroben auf Reisen schickt. Zirkulierendes bakteriologisches Wissen und die polnische Medizin 1885–1939 (Historische Wissensforschung, Bd. 9). *Tübingen: Mohr Siebeck, geb., 352 S., zahlr. Abb., 59,00 * *€, ISBN*:* 978-3-16-155064-5.*

**DOI:** 10.1007/s00048-021-00309-4

**Published:** 2021-07-23

**Authors:** Elzbieta Kassner

**Affiliations:** Leibniz Universität Hannover, Hannover, Deutschland

In ihrer als Buch erschienenen Dissertation über das zirkulierende bakteriologische Wissen um 1900 geht Katharina Kreuder-Sonnen einer originellen Frage nach: Wie schickte man in einem lokalen Labor beheimatete Mikroben auf Reisen? Ausgehend vom national und kulturell heterogenen, von der Medizin- und Wissenschaftshistoriografie kaum in den Blick genommenen Raum Ostmitteleuropa zeichnet sie die Verflechtungen und Netzwerke polnischsprachiger Wissenschaftler und Wissenschaftlerinnen in transnationalen Wissensräumen in der Zeit von 1885 bis 1939 nach. Die vermeintlich periphere Lage Polens bietet hierzu eine spannende Perspektive. Zentral geht es der Autorin darum, Grenzüberschreitungen der bakteriologischen Praxis von der Mikroebene aus im Konkreten und anhand ihrer Akteure nachzuvollziehen. Sie führt die Leserinnen und Leser in die Laborräume der großstädtischen Zentren von Paris und über Berlin nach Warschau, pendelt zwischen städtischen Metropolen und ländlicher Provinz, um von Lemberg aus schließlich Tunis und Addis Abeba zu erreichen. Dort betrachtet sie Praktiken der medizinischen Wissensproduktion zu Infektionskrankheiten. Angeleitet ist die Untersuchung von den Fragen danach, wie die Wissensbewegung in ihrer Dynamik fassbar gemacht werden kann, welche Anstrengungen unternommen wurden, um ein bakteriologisches Wissen zu produzieren und zirkulieren zu lassen und was benötigt wurde, um einem Wissensbestand internationale Anerkennung zu verschaffen und ihn an einem neuen Ort zu verankern.

Impulsgebend waren für Kreuder-Sonnen dabei die Arbeiten von Bruno Latour und die relationale Herangehensweise an die Kategorien des Lokalen, Transnationalen und Globalen. Für den Betrachtungszeitraum geht sie entlang des bisher kaum erforschten mehrsprachigen Quellenmaterials in die geografisch entlegenen Settings der Wissensproduktion. In Anlehnung an die Akteur-Netzwerk-Theorie zeigt sie die Vielfalt der Akteure, ihre Potenziale und Widerstände auf und untersucht, wie sie sich „selbst, andere oder anderes kontextualisieren und mit Sinn versehen“ (7). So werden der korrekt zubereitete bakteriologische Nährboden zur Kultivierung der Mikroben, das Laborequipment, die Fütterung von Kleiderläusen zu bedeutsamen Elementen transnational zirkulierenden Wissens.

Die Studie beginnt Mitte der 1880er Jahre in Warschau, wo der junge Mediziner Odo Bujwid nach Studienaufenthalten bei Pasteur und Koch den Versuch unternahm, Pariser Impfstoffanwendung und Berliner Labornetzwerke zu mobilisieren und die jeweiligen bakteriologischen Praktiken der polnischen *medical community* zu vermitteln. Eindrucksvoll zeichnet Kreuder-Sonnen die einzelnen Etappen der komplexen Laborprozesse nach, die zunächst in Text und Grafik, später mittels Mikrofotografien auf Papier gebannt wurden, so in Form „papierner Inskriptionen“ als Zeitschriftenaufsätze und Broschüren veröffentlicht und in die *medical community* transportiert werden konnten. Praxisorientierte Mediziner konnten bakteriologische Labors nur fragmentiert in den medizinischen Arbeitsalltag integrieren; erst der Aufbau eines öffentlichen Gesundheitswesens mit dem Staatlichen Hygieneinstitut als zentralem Akteur epidemiologischer Belange sollte der bakteriologischen Praxis zu einem breiten Aufschwung verhelfen. Als Verbündete des gesundheitspolitischen *state-building* fügte sich die Bakteriologie in die staatlichen Konzepte der Seuchenbekämpfung ein und wurde Teil einer Machttechnik, die Kranke wie Mikroben der Kontrolle durch den polnischen Staat zu unterwerfen suchte. Mit umfassendem Blick auf den Wirkungskreis unter anderem Marcin Kacprzaks, Ludwik Flecks und Ludwik Hirschfelds arbeitet Kreuder-Sonnen die Fragen und Entwicklungen um den bakteriologischen Denkstil heraus.

An der Entwicklungsgeschichte der Fleckfieberimpfung in den 1930er-Jahren macht die Autorin deutlich, dass auch mutmaßlich periphere Orte der Wissenschaft zu globalen Größen heranwachsen können. Konnte zwar die Gebundenheit der Fleckfieber-Wissensproduktion an die Orte der Epidemien mit der Übersetzung der Krankheit in „Labor-kompatible Formate“ von diesen abgekoppelt werden, so blieb die Impfstoffproduktion jedoch zunächst an die spezifische Laborinfrastruktur bei Lemberg gebunden. Rudolf Weigls Forschung zur Entwicklung eines wirksamen Vakzins baute auf den bereits gewonnenen Erkenntnissen eines internationalen Forschernetzwerks auf. Dank Helena Sparrows Expertise begann der Impfstoff dann zu zirkulieren, gelangte über das Institut Pasteur nach Tunesien, später nach Peking und wurde Teil des globalen Wissensraums. Die Impfstoffentwicklung war eng mit dem geopolitischen Standort und den kulturellen Aspekten bakteriologischer Forschung verbunden. Weigls Impfkampagne der Karpatenbevölkerung in Ostpolen wie auch Sparrows Arbeit in Tunesien fügen sich in koloniale Diskurse ein. Mit der Berücksichtigung polnischer Kolonialdiskurse in der Globalgeschichte der polnischen Wissenschaft wird von Kreuder-Sonnen in diesem Zusammenhang ein Forschungsdesiderat benannt.

Die Arbeit Kreuder-Sonnens stellt insgesamt einen ausgesprochen wichtigen Beitrag zur Beforschung des historischen Raums Ostmitteleuropa dar. Ihre fundierte transnationale Herangehensweise zeigt, wie notwendig und zielführend es ist, in einer globalen Wissensgeschichte der Bakteriologie die Kategorien Zentrum und Peripherie neu zu denken. *Wie man Mikroben auf Reisen schickt* ist daher fraglos eine wertvolle und ernst zu nehmende Erweiterung des medizin- und wissenschaftshistorischen Publikationskorpus.

